# Bioinformatics analysis of DNMT1 expression and its role in head and neck squamous cell carcinoma prognosis

**DOI:** 10.1038/s41598-021-81971-5

**Published:** 2021-01-26

**Authors:** Jili Cui, Lian Zheng, Yuanyuan Zhang, Miaomiao Xue

**Affiliations:** 1 Department of General Dentistry, The First Affiliated Hospital, Zhengzhou University, No. 1 Jianshe Road, Zhengzhou, 450052 Henan China; 2 Key Laboratory of Clinical Medicine, The First Affiliated Hospital, Zhengzhou University, Zhengzhou, 450052 Henan China; 3 Department of Oral and Maxillofacial Surgery, The First Affiliated Hospital, Zhengzhou University, Zhengzhou, 450052 Henan China

**Keywords:** Cancer, Gene regulation in immune cells

## Abstract

Head and neck squamous cell carcinoma (HNSCC) is the sixth most common type of malignancy in the world. DNA cytosine-5-methyltransferase 1 (DNMT1) play key roles in carcinogenesis and regulation of the immune micro-environment, but the gene expression and the role of DNMT1 in HNSCC is unknown. In this study, we utilized online tools and databases for pan-cancer and HNSCC analysis of DNMT1 expression and its association with clinical cancer characteristics. We also identified genes that positively and negatively correlated with DNMT1 expression and identified eight hub genes based on protein–protein interaction (PPI) network analysis. Enrichment analyses were performed to explore the biological functions related with of DNMT1. The Tumor Immune Estimation Resource (TIMER) database was performed to explore the relationship between DNMT1 expression and immune-cell infiltration. We demonstrated that DNMT1 gene expression was upregulated in HNSCC and associated with poor prognosis. Based on analysis of the eight hub genes, we determined that DNMT1 may be involved in cell cycle, proliferation and metabolic related pathways. We also found that significant difference of B cells infiltration based on TP 53 mutation. These findings suggest that DNMT1 related epigenetic alterations have close relationship with HNSCC progression, and DNMT1 could be a novel diagnostic biomarker and a promising therapeutic target for HNSCC.

## Introduction

Head and neck squamous cell carcinoma (HNSCC), an aggressive malignancy that is difficult to diagnose early, accounts for more than 95% of head and neck cancers^1,2^. Most HNSCC patients are diagnosed at an advanced stage with an extremely poor prognosis. It is estimated that 400,000 new patients are diagnosed annually worldwide^3^. Surgical resection, along with radiotherapy and chemotherapy, is recommended for early stage HNSCC. Although a comprehensive and multidisciplinary approach, including chemotherapy, radiotherapy, and immunotherapy, are used for advanced-stage HNSCC, the 5-year overall survival (OS) rate has remained below 50%^4,5^. Thus, by delineating the mechanisms of tumorigenesis and immune infiltration in HNSCC, a foundation for future therapeutic developments will be laid.

5-methylcytosine (m5C) modification is an important post-transcriptional methylation, and m5C is associated with the carcinogenesis and tumorigenesis of various cancers^6^. 5-methylcytosine regulators function as one kinds of important epigenetic modifiers^6^ . DNA methylation plays a role in epigenetic gene silencing, X chromosome inactivation, and genome stability^7^. It has also been shown that DNA methylation modifications are crucial for normal cell cycle regulation, development, and differentiation^8^. Recent studies have demonstrated that epigenetic DNA methylation alterations drive formation of many types of cancer^9^. DNA methylation is catalyzed by DNA methyltransferase enzymes (DNMTs), which regulate epigenetic modifications in mammals. DNMTs methylate CpG islands in promoters, aberrant modification of DNMTs methylate could cause carcinogenesis and tumor progression^10^. DNA cytosine-5-methyltransferase 1 (DNMT1) acts as a crucial epigenetic modifier by regulating DNA methylation of target genes, which involves in tumor suppressor gene expression^11^. DNMT1 gene expression is also subject to epigenetic regulation. Numerous studies have shown that DNMTI is highly upregulated in various cancers such as cholangiocarcinoma^12^, hematological malignancies^13^, pancreatic cancer^14^, breast cancer^15^. DNMTI is required for maintaining the cancer stem cell status^16,17^. In lung cancer patients, high levels of DNMT1 expression are associated with poor clinical prognosis^18^.However, it is also reported that upregulation of DNMT1 also functions in tumor-suppressor gene activation^19,20^. Further, DNMT1 regulates the immune system and is indispensable for the inhibitory function of Foxp3 + Treg cells^21^. In our previous study, we utilized the bioinformation analysis and validated the characters of m5C related regulators in HNSCC, and DNMT1 functioned as a key regulator in HNSCC progression^22^. We identified that DNMT1 might serve as a crucial m5C regulator in malignant activities of HNSCC, and increased DNMT1 expression was associated with patient’s mortality rate^22^. Nevertheless, the comprehensively biological activities of DNMT1 in HNSCC tumorigenesis and progression has not yet been clarified.

In this study, we used public databases and online analysis websites to unravel the role of DNMT1 in HNSCC tumorigenesis and progression. Furthermore we identified methylation markers with diagnostic potential, and identified potential targets for the treatment of HNSCC.

## Results

### DNMT1 gene expression and mutation distribution

**Figure 1:**
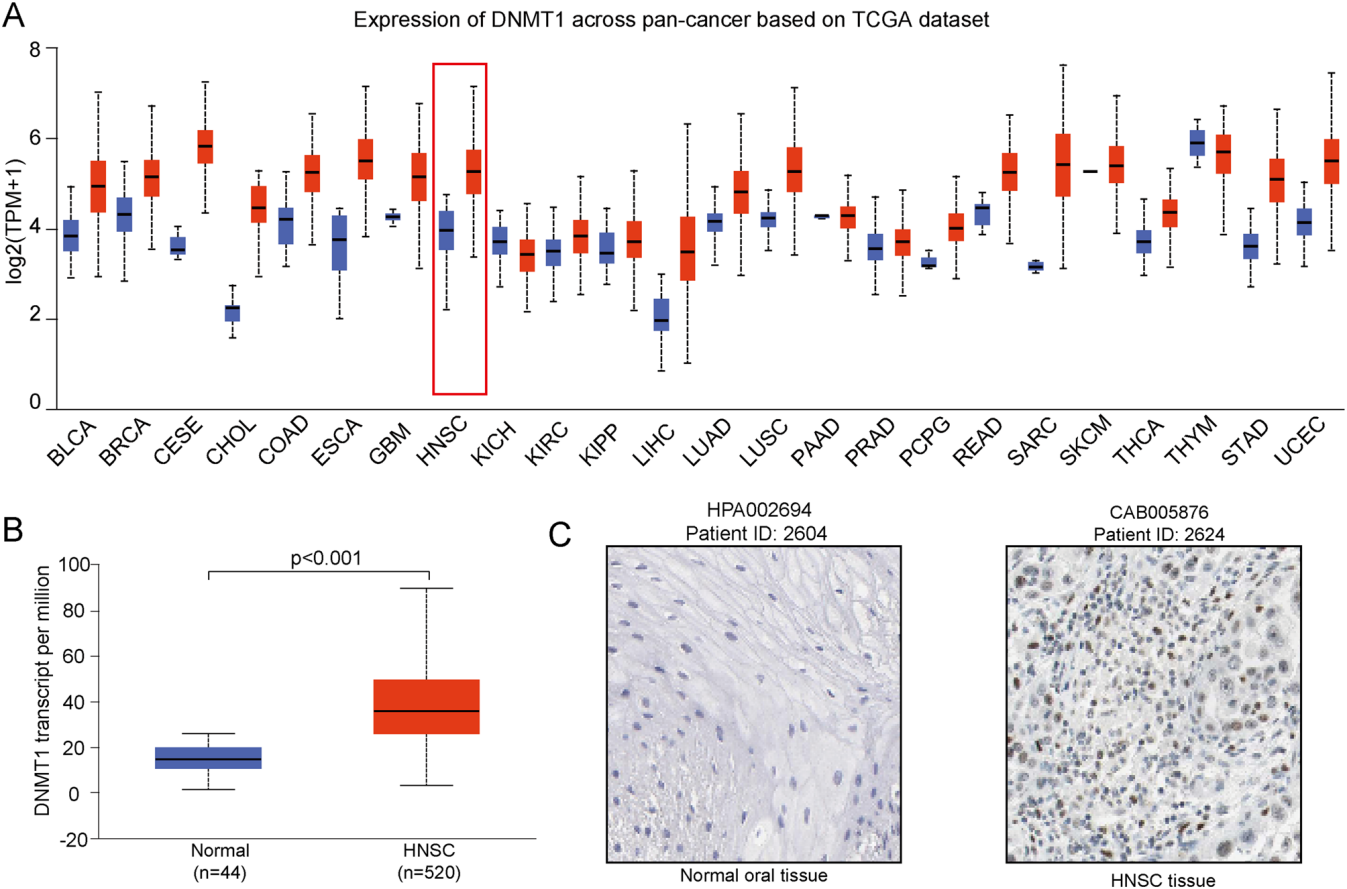
High DNMT1 expression was observed in head and neck cancers and in pan-cancers **(A)** High DNMT1 expression was found in several types of cancers. **(B)** The DNMT1 transcript level was significantly higher in HNSCC tissue than in non-tumor tissue. **(C)** DNMT1 protein expression was higher in HNSCC tumor tissue than in non-tumor tissue.

We performed a pan-cancer analysis of DNMT1 expression, and we found that DNMT1 was upregulated in several different tumor types (Fig. 1A). Specifically, transcription level of DNMT1 was significantly higher in HNSCC tumor tissues (n = 520) than in normal tissues (n = 44, *p* < 0.001) (Fig. 1B). To further validate the expression of DNMT1, the Immunohistochemistry (IHC) was applied to detect the protein expression of DNMT1 gene in normal tissues and tumor tissues based on HPA. Results showed that DNMT1 protein expression was significantly overexpressed in HNSCC tissues compared with normal tissue (Fig. 1C). Thus, DNMT1 had potential as a biomarker for diagnosis of HNSCC.

### The clinical features and prognosis of DNMT1 in HNSCC

**Figure 2:**
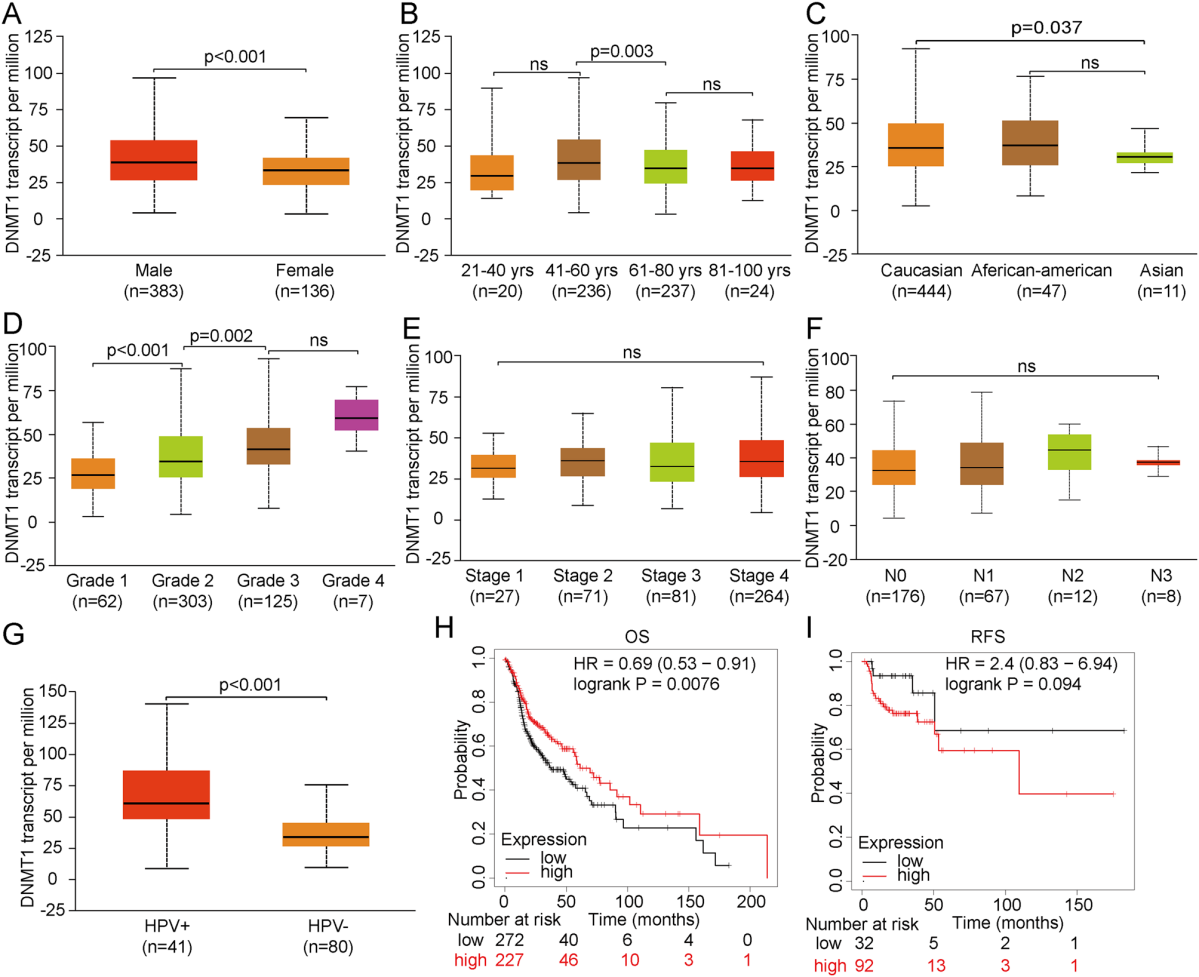
Relationships between DNMT1 expression and clinical characteristics and prognosis were analyzed. **(A–G)** DNMT1 expression correlated with gender, age, race, tumor grade, and HPV infectious status, but not with lymphatic invasion or tumor stage. **(H, I)** OS and RFS analyses demonstrated that patients with high DNMT1 expression had poor clinical prognosis.

To explore the DNMT1 related clinical characters, we determined whether gender, age, race, tumor stage, tumor grade, nodal metastasis, and human papillomavirus (HPV) infection status correlated with DNMT1 expression in HNSCC. We found that DNMT1 expression level was significantly higher in males patients than in females patients (*p* < 0.001) (Fig. 2A) and in the less than 60-year-old subgroups (n = 256), DNMT1 expression level was remarkably higher compared with over61-year-old subgroups (n = 261, *p* = 0.003) (Fig. 2B). We also observed that DNMT1 expression was higher in Caucasians compared with Asians and African-American subgroups (*p* = 0.037) (Fig. 2C). Advanced-grade tumors had higher DNMT1 gene expression level compared with lower grade tumor samples (Fig. 2D), but TNM stages and lymphatic invasion did not correlate with DNMT1 expression (Fig. 2E,F). We validated that HPV-positive patients (n = 41) had higher DNMT1 expression compared with HPV negative patients (n = 80) (*p* < 0.001) (Fig. 2G). Furthermore, we explored the overall survival (OS) and relapse-free survival (RFS) of HNSCC patients (Fig. 2H,I) and found that patients with high DNMT1 expression had better OS rate (HR = 0.69, logrank *p* = 0.0076). Patients with high expression level of DNMT1 showed no significant difference in RFS compared with low DNMT1 expression subgroups (HR = 2.4, logrank *p* = 0.094). DNMT1 may be potential genes for HNSCC poor prognosis prediction and may provide reference value for HNSCC treatment in the further.

### DNMT1 gene mutations and co-expressed genes

**Figure 3:**
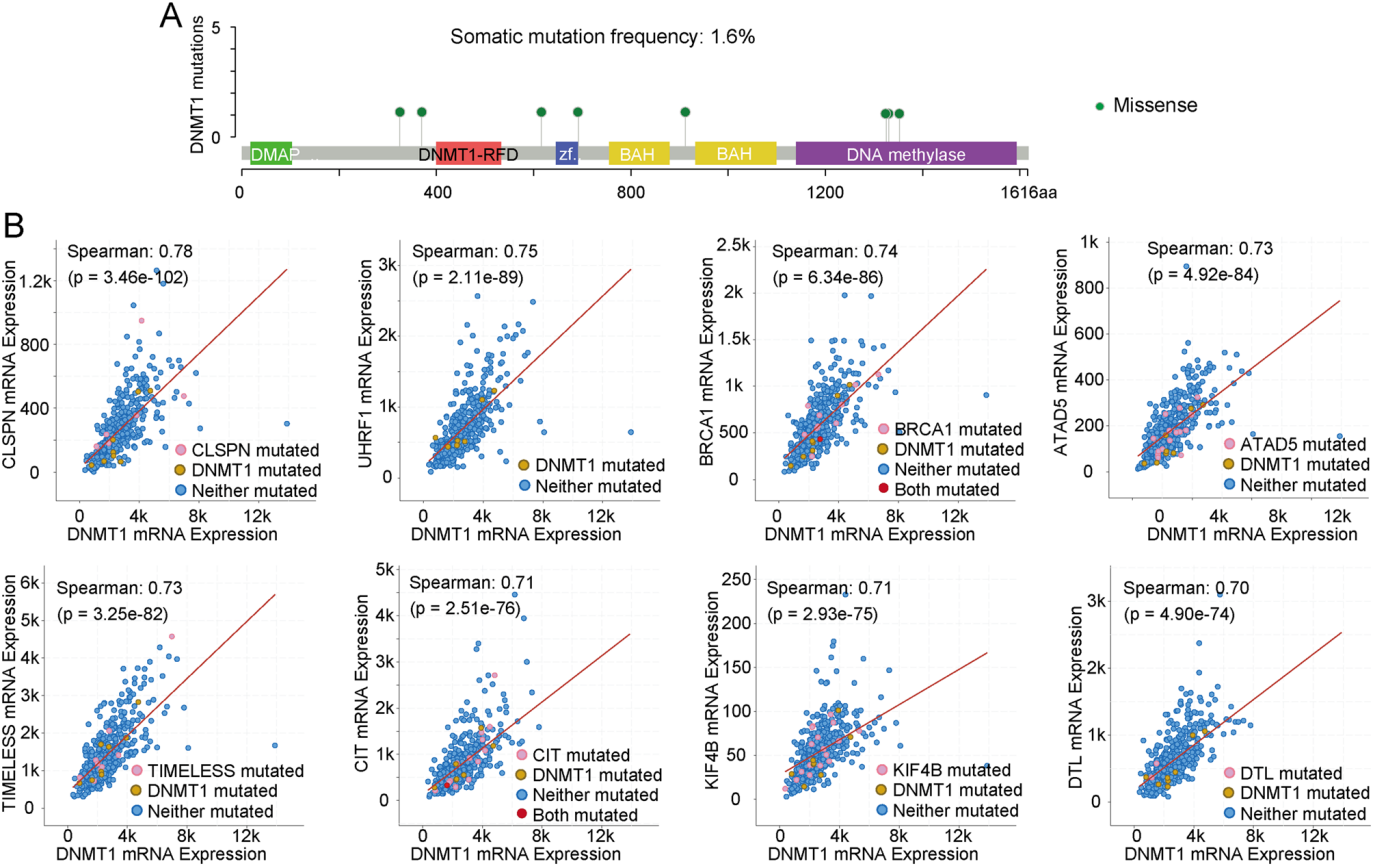
Distribution of DNMT1 mutations and co-expressed genes. **(A)** In HNSCC, the somatic mutation rate was 1.6%. **(B)** CLSPN, UHRF1, BRCA1, ATAD5, TIMELESS, CIT, KIF4B, and DTL genes were co-expressed with DNMT1.

The somatic mutation rate of DNMT1 in HNSCC patients was 1.6%, and the eight mutations identified were all missense mutations, P1330S, P1325S, E912Q, S1352G, P692S, H370Y, T616M, and R325L (Fig. 3A). We also identified eight co-related genes such as CLSPN, UHRF1, BRCA1, ATAD5, TIMELESS, CIT, KIF4B and DTL (Table 1).

**Table 1:** The co-expression genes of DNMT1 in HNSCC.

Correlated Gene	Cytoband	Spearman’s correlation	p-value
CLSPN	1p34.3	0.783	3.46E−102
UHRF1	19p13.3	0.75	2.11E−89
BRCA1	17q21.31	0.741	6.34E−86
ATAD5	17q11.2	0.735	4.92E−84
TIMELESS	12q13.3	0.73	3.25E−82
CIT	12q24.23	0.711	2.51E−76
KIF4B	5q33.2	0.707	2.93E−75
DTL	1q32.3	0.703	4.90E−74

The correlation between DNMT1 mutation status and the 8 genes mutation status were evaluated. In this study, the 8 genes showed significantly strong correlation with DNMT1 expression, as illustrated in Fig. 3B. CLSPN (Spearman: 0.78, *p* = 3.46e−102), UHRF1 (Spearman: 0.75, *p* = 2.11e−89), BRCA1(Spearman: 0.74, *p* = 6.34e−86), ATAD5 (Spearman: 0.73, *p* = 4.92e−84), TIMELESS (Spearman: 0.73, *p* = 3.52e−82), CIT (Spearman: 0.71, *p* = 2.51e−76), KIF4B (Spearman: 0.71, *p* = 2.93e−75), and DTL (Spearman: 0.70, *p* = 4.90e−74).

### DNMT1 expression associates with immune cell infiltration and HNSCC prognosis

**Figure 4:**
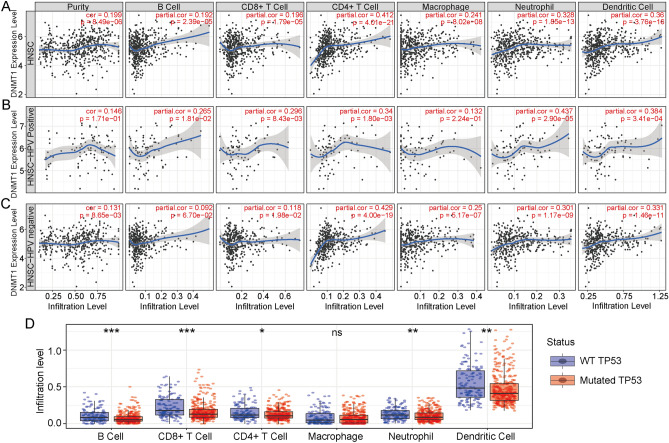
The relationship between DNMT1 expression and immune cell infiltration and clinical prognosis of HNSCC. **(A)** DNMT1 gene expression positively correlated with tumor purity and infiltration of B cells, CD8 + T cells, CD4 + T cells, macrophages, neutrophils, and dendritic cells in HNSCC. **(B)** DNMT1 expression did not correlate with tumor purity and macrophage infiltration in the HPV-positive HNSCC group. Infiltration of B cells, CD8 + T cells, CD4 + T cells, neutrophils, and dendritic cells correlated weakly with DNMT1 expression in the HPV-positive HNSCC subgroup. **(C)** DNMT1 expression positively correlated with tumor purity and infiltration of CD8 + T cells, CD4 + T cells, macrophages, neutrophils, and dendritic cells, but not with infiltration of B cells, in the HPV-negative HNSCC subgroup. **(D)** TP53 mutation with immune cells infiltration in HNSC.

Tumor-infiltrating immune cells are an independent predictor of cancer therapy and prognosis. We investigated the correlation between DNMT1 expression and infiltration of various immune cells in HNSCC, and we found that DNMT1 expression weakly correlated with immune cell purity (Correlation = 0.199, *p* = 8.49e−06). And the top three immune cells that correlated with DNMT1 expression in HNSCC were CD4 + T cells (Cor = 0.412), neutrophils (Cor = 0.328), and dendritic cells (Cor = 0.360) (Fig. 4A). To further investigate associations with immune-cell infiltration status, HNSCC patients were divided into HPV-negative and HPV-positive subgroups. The results illustrated that HPV-negative subgroup had mildly correlation with immune purity and immune cells infiltration such as CD4 + T cells (Cor = 0.429, *p* = 4.00e−19), macrophage (Cor = 0.25, *p* = 5.17e−07), neutrophil (Cor = 0.301, *p* = 1.17e−09), dendritic cell (Cor = 0.331, *p* = 1.46e−11) (Fig. 4B,C). Furthermore, we identified the immune cells infiltration with TP53 mutation status, the results demonstrated that the TP53 mutation had significant difference in B cells, CD8 + Tcells, neutrophil, dendritic cells infiltration, and weak difference in CD4 + T cells infiltration (Fig. 4D). We also validated the correlation between the immune-cell infiltration status and prognosis in HNSCC. In this study, we demonstrated that the cumulative survival in B cell high expression group showed weak difference compared with low expression group (Log-rank *p* = 0.045) (Fig. S1a). In HPV-positive group, immune cells high expression groups had better cumulative survival compared with low expression patients, such as in B cell (Log-rank *p* = 0.013), CD8 + T cell (Log-rank *p* = 0.015), neutrophil (Log-rank *p* = 0.074) (Fig. S1b). However, in HPV-negative group the cumulative survival in different immune cells infiltration showed no significantly difference (Fig. S1c). In this part, our finding indicated that DNMT1 expression have closely relationship with immune cells infiltration.

### Identification and analysis of DEGs

**Figure 5:**
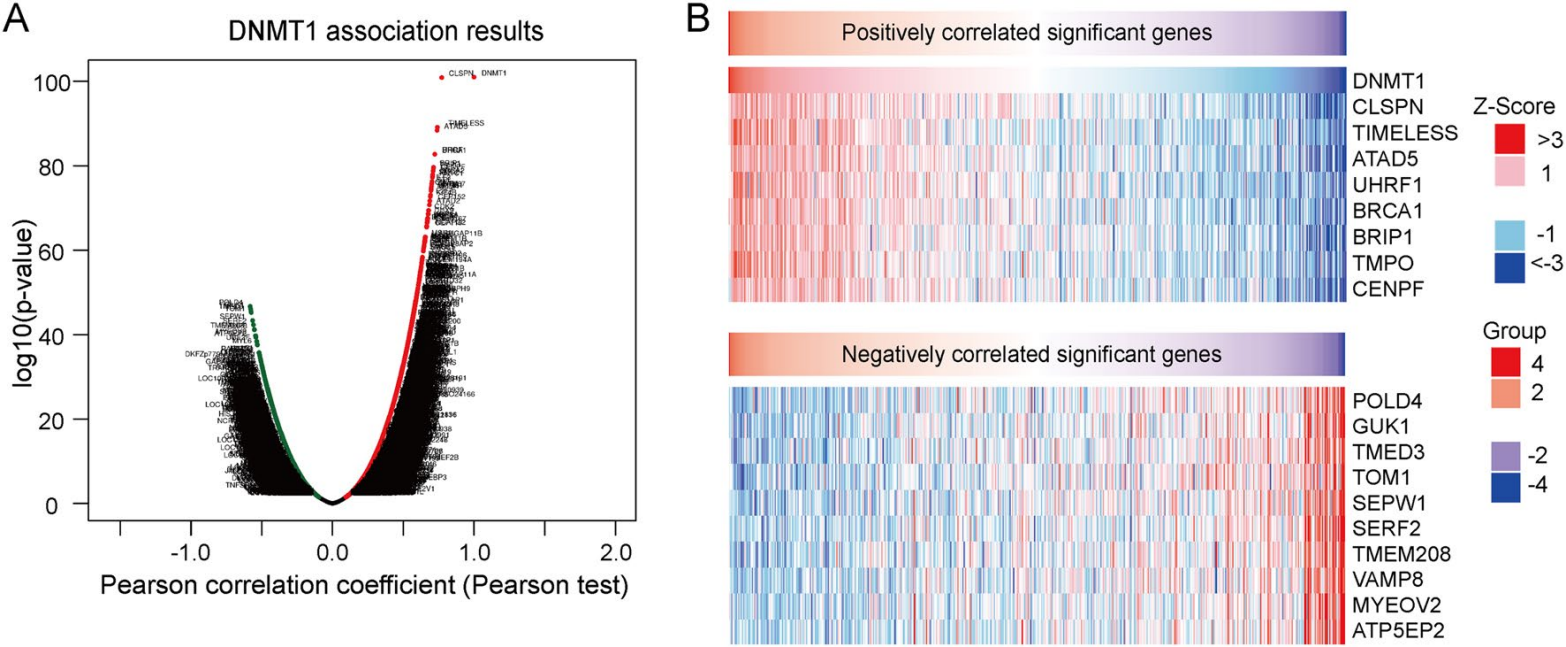
Distribution of genes that associated with DNMT1 expression. **(A)** The volcano map shows the genes that positively and negatively correlated with DNMT1 expression. **(B)** The heat-map shows the top 10 genes that positively correlated and negatively with DNMT1 expression.

To explore the DNMT1 related differently expressed genes, we identified 100 positively correlated and 100 negatively correlated genes as illustrated in Fig. 5A. To clarify the top correlated genes, we mapped the top 8 genes such as CLSPN, TIMELESS, ATAD5, UHRF1, BRCA1, BRIP1, TMPO, and CENPF, which were positively correlated with DNMT1. POLD4, GUK1, TMED3, TOM1, SEPW1, SERF2, TMEN208, VAMP8, MYEVO2, and ATP5EP2 were top 10 significantly negative correlated genes with DNMT1 expression (Fig. 5B), and the map showed that DNMT1 expression associated with multiple genes expression.

### Enrichment analysis of DNMT1-related genes

**Figure 6:**
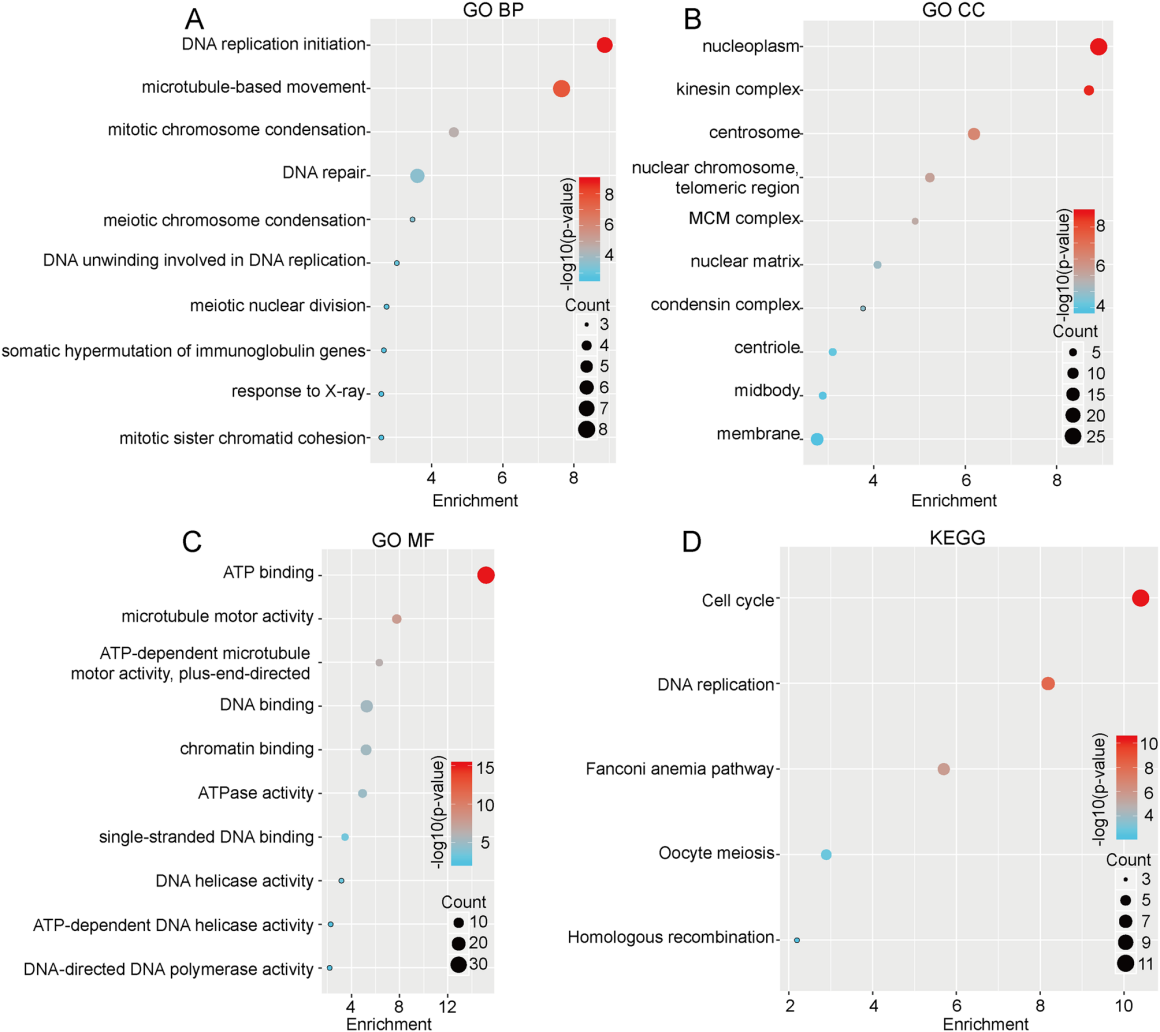
GO enrichment and KEGG pathways analyses of the top 100 genes that positively correlated with DNMT1 expression. **(A)** GO BP enrichment analysis of DNMT1-associated genes. **(B)** GO CC enrichment analysis of DNMT1-associated genes. **(C)** Go MF enrichment analysis of DNMT1-associated genes. **(D)** KEGG analysis of DNMT1-associated genes.

To identify the pathways that the DNMT1-related genes are involved in, we performed GO and KEGG analyses. As shown in Fig. 6A, The GO BP enrichment results showed that the DNMT1-related genes associate with DNA replication, transcription initiation, and microtubule-based movement. GO CC analysis showed a significant association with nucleoplasm activity (Fig. 6B), and GO MF analysis showed a strong association with ATP binding (Fig. 6C). Furthermore, KEGG results showed remark associations with the cell cycle and DNA replication (Fig. 6D). Overall, DNMT1 appears to function in basic cellular activities such as, DNA replication initiation, nucleoplasm, ATP binding, and cell cycle.

### PPI network and hub gene selection of DNMT1

**Figure 7:**
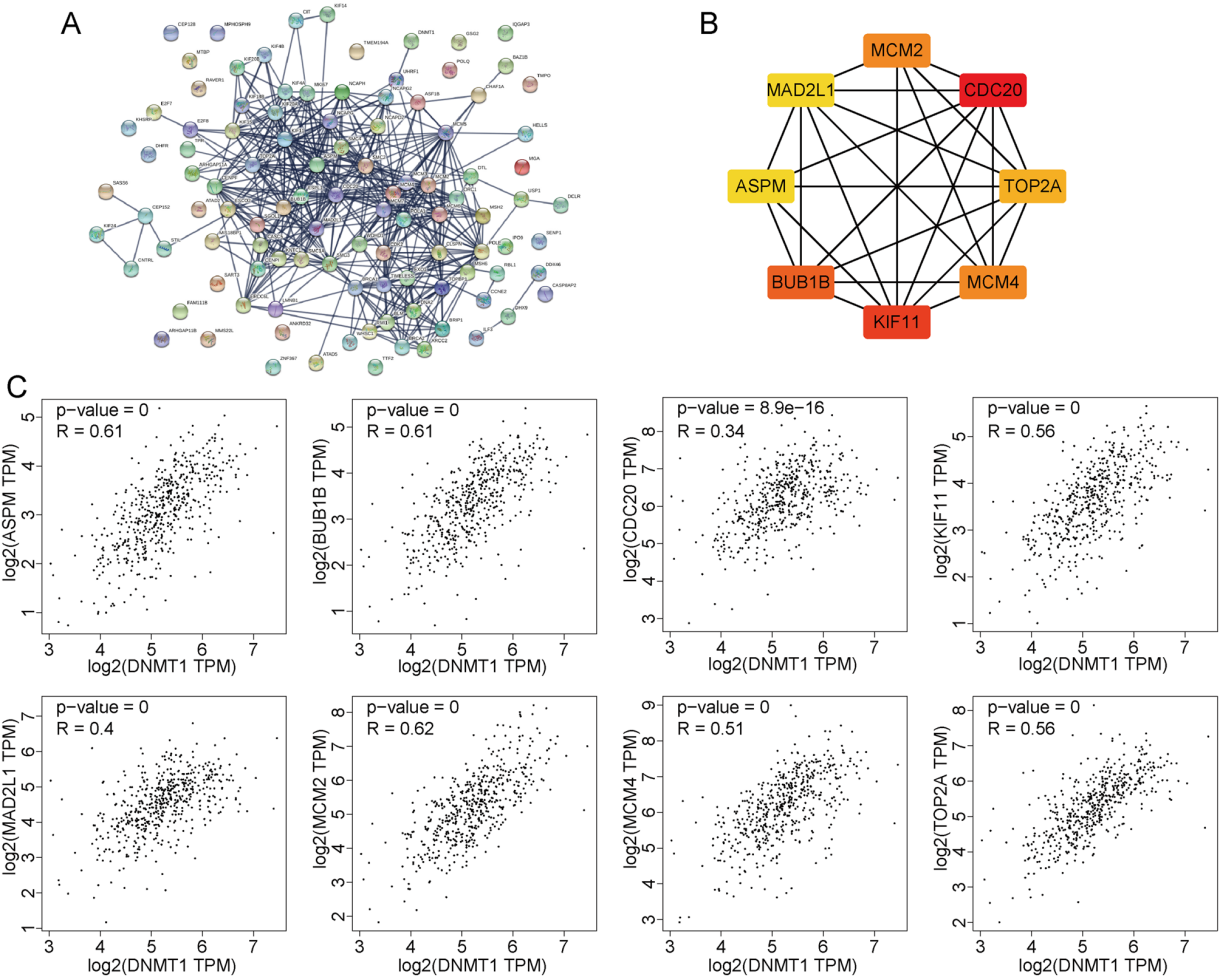
The PPI network was based on the top 100 DNMT1-related genes. **(A)** The distribution of the top 100 DNMT1-related genes is shown, and the red-colored nodes represent the log (FC) values of the Z-scores of the proteins that interact with the designated protein. **(B)** 8 hub genes were identified with Cytoscape software. The red-color represents genes that positively correlated with DNMT1 expression. **(c)** Correlations between expression of hub genes and DNMT1 expression.

To further investigate the function of DNMT1 in HNSCC, we constructed a PPI network based on the top 100 positively correlated DNMT1-related genes, and we found that the top 100 positive DNMT1-related genes correlated with each other (Fig. 7A). We identified eight hub genes such as BUB1B, KIF11, CDC20, MCM2, MCM4, TOP2A, ASPM and MAD2L1 (Fig. 7B) and we detected the correlations between hub genes and DNMT1 expression (Fig. 7C). Moreover, we found that expression levels of all eight hub genes were significantly increased in HNSCC tissue compared with normal tissue (all *p* < 0.001) (Fig. S2a). To determine the overall survival among these 8 genes, we applied Kaplan–Meier analysis, and results showed that high expression level of ASPM in HNSC had better prognosis (HR = 0.71 (0.51–0.98), logrank *p* = 0.035). The low expression level of BUB1B in HNSCC was associated with poor prognosis (HR = 1.38 (0.94–1.78), logrank *p* = 0.046) (Fig. S2b). Thus, the hub genes have potential as novel targets for delineation of the tumor progression mechanism in HNSCC.

## Discussion

HNSCC has a high mortality rate and is difficult to detect at an early stage due to the lack of effective early monitoring and screening factors^2^. Once HNSCC is diagnosed, it is already advanced and the prognosis is extremely poor. It has been found that traditional surgery, postoperative radiotherapy, and chemotherapy have not significantly improve survival of advanced HNSCC^1^. The recent emergence of immunotherapy may ultimately improve the prognosis of advanced-stage HNSCC patients, but substantial research is still needed.

The epigenetic alterations contain the reversible DNA methylation and histone acetylation or methylation^23^. DNA methylation modification is an important regulator of cell activity, and abnormal regulation due to alterations in methylation can lead to tumorigenesis^15^. DNMTs are involved in initiation of mammalian chromatin remodeling and gene expression regulation in diverse physiological and pathophysiological processes^23,24^. m5C functions as an important epigenetic modifier in DNA and RNA with transcriptional expression^25^. m5C plays vital roles in HNSCC carcinogenesis and tumor progression^22^. In addition, genomic alterations in m5C-related modifiers have close relationship with clinicopathological characteristics. In our previous study, we found that the DNMT1, a key m5C regulator, was closely related with HNSCC prognosis, and also identified the DNMT1 as death risk predictor in HNSCC progression.

DNMT1 is an important regulator of methylation, since it participates in the regulation of various key biological activities and the immune cell micro-environment^26,27^. However, the biological roles and potential mechanisms of DNMT1 expression remain largely unknown in HNSCC. Insights into the mechanism of DNMT1 expression will provide a foundation to determine the role of DNMT1 in HNSCC and may lead to improvements in the clinical management and diagnosis of HNSCC patients.

It has been shown that dysregulation of DNMT1 associates with tumorigenesis and progression of multiple types of cancer. Overexpression of DNMT1 has been observed in macroadenomas, invasive tumors, and advanced pituitary adenoma^28^. DNMT1 has been shown to be involved in drug resistance of non-small cell cancer cells, and this drug resistance was reversed upon treatment with a micro-RNA that negatively regulates DNMT1 expression^29,30^. Similarly, increased DNMT1 expression has been found in cancerous mammary gland tissues of a mammary cancer animal model. DNMT1 is also expressed highly in diffuse large B-cell lymphoma^31^. Based on previous bioinformation analysis of HNSCC and m5C regulators, we comprehensively explored the m5C regulator DNMT1 and its roles in HNSCC carcinogenesis and progression process. Consistent with previous studies, we also found that DNMT1 is expressed highly in HNSCC tissue compared with normal tissue. Pan-cancer analysis also showed upregulation of DNMT1 transcription in several cancer types.

We found that high DNMT1 expression associated with HNSCC patients ≥ 60 years old, which is consistent with a previous study in which high DNMT1 expression was observed in peripheral blood mononuclear cells (PBMCs) from patients up to 64 years old in a large European cohort^32^. Previous studies have shown that genetic alterations in DNMT1 associated with a low risk of developing breast cancer in the European Caucasian population and that DNMT1 expression was higher in Caucasians than in Asians and African-Americans^33^. We also demonstrated that HPV-positive HNSCC was more malignant and associated with poor prognosis. DNMT1 gene expression was also positively correlated with poor prognosis, suggesting that DNMT1 expression could be a potential biomarker of HNSCC prognosis. The somatic mutation rate of DNMT1 was 1.6%, and all of the mutations were missense mutations. Our results demonstrated that DNMT1 mutations may cause genomic instability leading to tumorigenesis.

TP53 is a suppressor gene with high frequency of mutation in tumors, and has become an independent prognostic factor in tumorigenesis. TP53 mutation can significantly activate immune checkpoints, initiate effector T cells and increase immune factors expression levels^34^. In lung cancer immunotherapy, TP53 mutation can be recognized as a predictor of immunotherapy sensitivity^35^. However, TP53 mutational status based immune responses and the potential clinical characters involves in HNSCC has been evaluated.

Therefore, it is necessary to further investigate the meaningful immune-related prognostic models and immune-related effects of TP53 status to provide powerful prognostic biomarkers and increase the effectiveness of immunotherapy^36^. Recent studies have shown that immune-cell infiltration contributes to the tumor immune micro-environment, which affects immunotherapy^37,38^. In this study, we demonstrated that HNSCC patients with abundant infiltration of immune cells, such as B cells, CD8 + T cells, neutrophils, had better prognosis. Previous studies have shown that high levels of infiltration by CD8 + tumor-infiltrating lymphocytes and B cells led to better clinical outcomes and prognosis. Therefore, the immune-cell landscape of HNSCC may associate with HNSCC clinical parameters.

In our study, DNMT1 was upregulated in various cancers, especially in HNSCC tumor tissues compared with adjacent tissues. And DNMT1 expression was much higher in male patients, and higher in elder patients over 60-year old. The advanced stages of HNSCC had higher DNMT1 transcription levels compared with early stages. Furthermore, we also investigated the DNMT1 related co-expression genes, such as CLSPN, UHRF1, BRCA1, ATAD5, TIMELESS, CIT, KIF4B and DTL. In addition, the associated top 3 immune cells infiltrations of DNMT1 were as follows, CD4 + T cells (Cor = 0.412), neutrophils (Cor = 0.328), and dendritic cells (Cor = 0.360). We also comprehensively explored the distribution of DNMT1 related DGEs, pathways enrichment analysis and the PPI network in HNSCC. Therefore, we identified DNMT1 as a potential biomarker for HNSCC. Further studies are needed to uncover the underlying mechanism of DNMT1 in tumorigenesis to develop biomarkers for HNSCC early detection and prognosis.

## Material and methods

### Clinical datasets and DNMT1 expression

The Cancer Genome Atlas (TCGA) database provide comprehensive molecular characterizations of multiple cancers and comprehensive data on gene profiling and sequencing, as described^39–42^. These bioinformatic computational tools were used to identify novel therapeutic and diagnostic biomarkers among 520 HNSCC tumor tissues and 44 adjacent tissues. The gene transcription profiles and clinical information f or these tissue samples were included in this study. Samples from patients with incomplete information were excluded. The Ualcan database (http://ualcan.path.uab.edu/analysis.html) is a web-portal platform to further explore TCGA gene expression data^43^. Pan-cancer analysis of DNMT1 expression and its relationship with general clinical information were performed via Ualcan. We also used Human Protein Atlas (HPA, http://www.proteinatlas.org/), a free and open source website used to investigate protein alterations and expression.

DNMT1 mutation and co-expression information was obtained from the cbioportal database (https://www.cbioportal.org/), and the Linkomics database (http://linkedomics.org/) was used to identify differentially expressed genes (DEGs) of DNMT1^44,45^.

### Tumor immune estimation resource (TIMER) database

The TIMER database^46^ (Version 1.0 1, https://cistrome.shinyapps.io/timer/) is a public database used to explore the relationship between cancer and immune-cell infiltration. TP53 mutation appears to be the most frequent genetic alteration overall. In order to clarify the immune cells infiltration with TP53 mutation in HNSCC, we utilized TIMER to evaluate the impact of immune factors and DNMT1 on HNSCC prognosis and to evaluate the effect of TP53 gene expression on immune cell infiltration.

### Survival of HNSCC patients

The Kaplan–Meier plotter online tool^47^ (https://kmplot.com/analysis/) was used to assess the correlation between DNMT1 gene expression and HNSCC survival. Statistical significance was determined by hazard ratio (HR) with a 95% confidence interval (CI) and log-rank *p*-value.

### Protein–protein interaction (PPI) network analysis and hub gene selection

LinkedOmics (http://linkedomics.org/) was also performed to identify correlated genes^44^. A gene activity network was constructed with the top 100 genes that positively correlated with DNMT1 expression. The top eight genes that positively correlated with DNMT1 expression were used in enrichment analyses for hub genes and the protein–protein interaction (PPI) network. The PPI network of overlapped DEGs was constructed by STRING and visualized with Cytoscape. The method has been described in previous study^48^.

### Gene function enrichment analyses

GO (Gene Ontology) and KEGG (Kyoto Encyclopedia of Genes and Genomes) pathway enrichment analyses were used to evaluate function. GO analysis assesses biological processes (BP), molecular functions (MF), and cellular components (CC). KEGG pathway enrichment analysis was performed using the online website DAVID as described in previous study^49^.

### Statistical analysis

The model construction and statically methods have been described as previous studies^50–52^. The absolute values 0.00–0.19, 0.20–0.39, 0.40–0.59, 0.60–0.79, and 0.80–1.0 indicated very weak, weak, moderate, strong, and very strong correlations, respectively. *p* < 0.05 was considered statistically significant.

## Supplementary Information

Supplementary Figure S1.

Supplementary Figure S2.

Supplementary Legends.
