# Supplementary figures and images for: Bioinformatics analysis of DNMT1 expression and its role in head and neck squamous cell carcinoma prognosis
Source: Sci Rep. 2021 Jan 26;11:2267. doi: 10.1038/s41598-021-81971-5 (PMC7838186; doi:10.1038/s41598-021-81971-5)

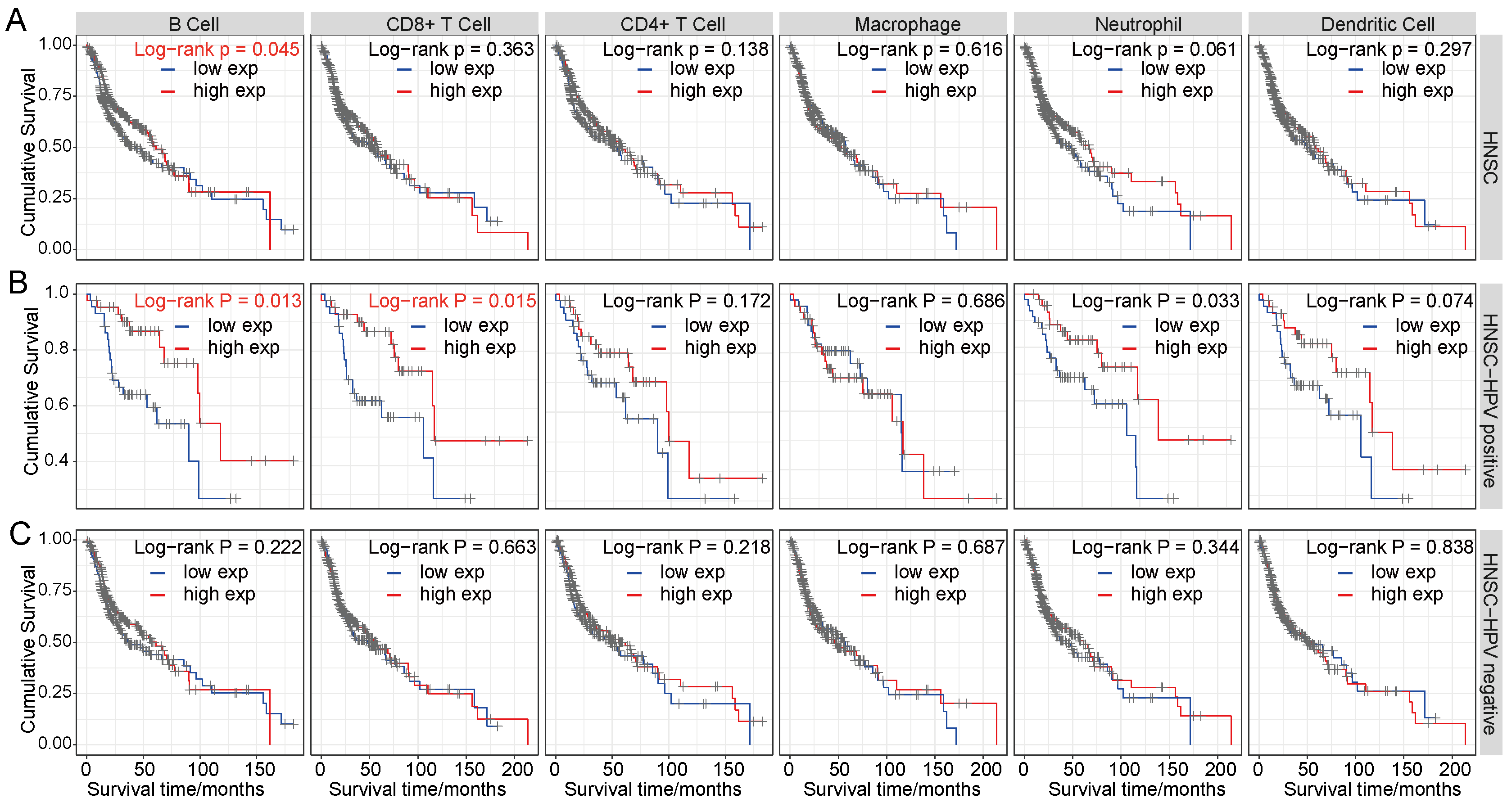

Supplement: Supplementary file 1 — Supplementary Figure S1. [file 41598_2021_81971_MOESM1_ESM.tif]

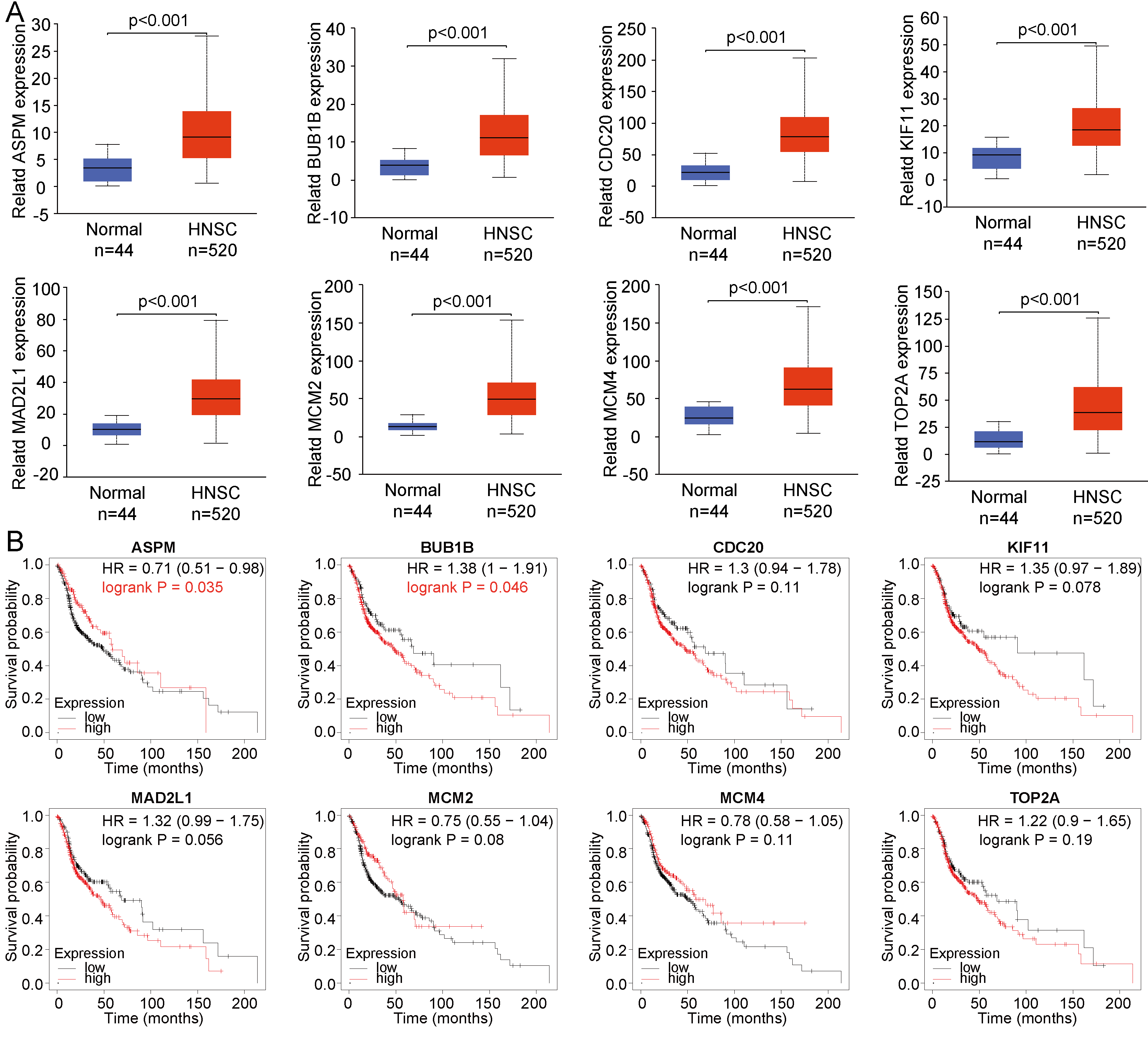

Supplement: Supplementary file 2 — Supplementary Figure S2. [file 41598_2021_81971_MOESM2_ESM.tif]
