# Supplementary material for: Bioinformatics analysis of DNMT1 expression and its role in head and neck squamous cell carcinoma prognosis
Source: Sci Rep. 2021 Jan 26;11:2267. doi: 10.1038/s41598-021-81971-5 (PMC7838186; doi:10.1038/s41598-021-81971-5)
Supplement: Supplementary file 3 — Supplementary Legends. [file 41598_2021_81971_MOESM3_ESM.docx]

**Bioinformatics analysis of DNMT1 expression and its role in head and neck squamous cell carcinoma prognosis**

**Jili Cui^1,2^**†**, Lian Zheng^2,3^**†**, Yuanyuan Zhang^1,2*^, Miaomiao Xue^1,2*^**

^1^Department of General Dentistry, the First Affiliated Hospital of Zhengzhou University, Zhengzhou, Henan 450052, China

^2^Key Laboratory of Clinical Medicine, the First Affiliated Hospital of Zhengzhou University, Zhengzhou, Henan 450052, China

^3^Department of oral and maxillofacial surgery, the First Affiliated Hospital of Zhengzhou University, Zhengzhou, Henan 450052, China

† Jili Cui and Lian Zheng contributed equally to this work.

***Correspondence Author:**

**Miaomiao Xue**, department of General Dentistr, the First Affiliated Hospital of Zhengzhou University, NO.1 Jianshe Road, Zhengzhou, Henan, 450052; E-mail: mmxue001@163.com

**Yuanyuan Zhang**, department of General Dentistr, the First Affiliated Hospital of Zhengzhou University, NO.1 Jianshe Road, Zhengzhou, Henan, 450052; E-mail: zhyy143@126.com

**Figure S1.** Immune cells infiltration associated with HNSC patient’s prognosis. (a), Kaplan-Meier survival curves that compare high and low levels of immune cell infiltration. (b), Survival curves that compare high and low levels of immune cell infiltration in the HPV-positive HNSCC subgroup. (c), Survival curves that compare high and low levels of immune cell infiltration levels in the HPV-negative HNSCC subgroup.

**Figure S2.** Expression of hub genes and HNSCC prognosis. (a), Expression of eight hub genes in normal and tumor tissues. (b), Associations of the expression of the eight hub genes with OS and HNSCC prognosis.
